# Adenoviral infectivity of exfoliated viable cells in urine: Implications for the detection of bladder cancer

**DOI:** 10.1186/1471-2407-11-168

**Published:** 2011-05-12

**Authors:** Anuradha Murali, Laura Kasman, Christina Voelkel-Johnson

**Affiliations:** 1 Department of Microbiology & Immunology, Medical University of South Carolina, Charleston SC, 29425, USA

## Abstract

**Background:**

Bladder cancer, the 5^th ^most common malignancy in the USA, is often detected as a result of incidental findings or by presenting hematuria. Once diagnosed the disease is one of the costliest cancers to treat due to frequent, invasive and often lifelong follow-up procedures. Because cells are shed into urine, there has been an emerging effort to develop non-invasive tests for the detection of bladder cancer. Expression of survivin, a member of the inhibitor of apoptosis protein family, has been associated with bladder cancer. Therefore, the goal of this study was to determine the feasibility of transducing viable exfoliated cells obtained from urine with an adenoviral vector in which a reporter gene is under the control of the survivin promoter.

**Methods:**

Exfoliated cells from urine were obtained from 36 human subjects (> 40 years old). An adenovirus in which GFP expression is under control of the survivin promoter (Ad.Surv.GFP) was generated. An adenovirus in which GFP is expressed from the CMV promoter served as a control. GFP expression was analyzed by fluorescent microscopy and quantified by flow cytometry.

**Results:**

Short-term cultures from exfoliated cells in urine could be established in 16 of 31 samples. These cultures were successfully transduced with Ad.CMV.GFP. Analysis of GFP expression following transduction with Ad.Surv.GFP, indicated that the survivin promoter was preferentially active in UM-UC-3 bladder cancer cells compared to non-malignant UROtsa cells. Interestingly, baseline levels of GFP expression in cultures from exfoliated cells in urine exhibited higher baseline levels than UROtsa following transduction with Ad.Surv.GFP.

**Conclusions:**

We demonstrated the feasibility of establishing and analysing short-term cultures isolated from exfoliated cells in voided urine by means of adenoviral transduction, thereby forming the foundation for future studies to determine the specificity and sensitivity of a non-invasive test based on survivin promoter activity.

## Background

According to the American Cancer Society bladder cancer is the 5th highest in estimated new cases of cancers by site with 14,680 bladder cancer deaths and 70,530 new diagnoses in 2010 [1,2]. Bladder cancer can be categorized into non-muscle-invasive bladder cancer or muscle-invasive bladder cancer where 80% of the newly diagnosed cancers are non-muscle-invasive bladder cancer. Unfortunately, 70% of the patients will have recurrence of the disease and 10-30% will progress to muscle-invasive disease. Bladder cancer is detected as a result of incidental findings or by presenting hematuria. While hematuria is associated with benign conditions such as urinary tract infections and urolithiasis, 10% of the patients with gross hematuria are diagnosed with bladder cancer [3]. Contrary to these statistics, microhematuria is detected in 9 to 18% of normal individuals and 2-5% of patients with microscopic hematuria are diagnosed with bladder cancer. These findings support the need for non-invasive methods to detect bladder cancer prior to the onset of clinical symptoms.

Currently, fluorescence in situ hybridization (FISH), cystoscopy and cytology are methods used for bladder cancer surveillance in clinical practice. Patients diagnosed with non-invasive bladder cancer are subjected to repeat cystoscopy and cytology every 3 months for a minimum of 2 years. Cystoscopy is associated with severe discomfort and morbidity of patients, resulting in poor patient compliance. Moreover, cystoscopy can miss the diagnosis of flat tumors or carcinoma-in-situ (CIS) leading to 10-30% false-negative results [4]. Based on recent cost-effectiveness studies, overall specificity for common urine-based tumor markers (bladder tumor antigen and nuclear matrix protein 22) was 73% to 90% and sensitivity was 49% to 77% [5]. The majority of bladder cancers is detected at an early stage and is treated by surgical resection followed by intravesical immunotherapy with Bacille Calmette-Guerin (BCG) occasionally in combination with interferon-α2b. However, response to BCG therapy is variable. While BCG therapy is the best available treatment, it can be limited by severe side effects, which results in early termination of treatment and reduced efficacy. The paucity of urine markers of bladder cancer with high specificity and sensitivity warrants identification of non-invasive screening methods for early detection and prognosis of bladder cancer.

Although to date, several markers of bladder cancer have been reported, such as nuclear matrix protein 22, hyaluronic acid, hyaluronidase and nuclear matrix proteins, these markers are ineffective in reducing the number of surveillance cystoscopies due to limited sensitivity and specificity [6]. Survivin is a member of the inhibitor of apoptosis protein (IAP) family and has been identified as a potential marker for detecting high-grade urothelial bladder cancer with 83% sensitivity and 88% specificity [7]. Cancer cells have the ability to evade apoptosis by up-regulating IAPs such as survivin and recent evidence suggests that urine survivin can be used as a diagnostic test for bladder cancer [8]. Moreover, measurement of mRNA levels suggests that the survivin promoter is active in malignant cells. An adenovirus in which a luciferase reporter is under control of the survivin promoter has been generated [9]. Taken together, promoter strength and the cancer-specificity of survivin indicate the possibility to utilize a survivin-driven reporter gene such as GFP (green fluorescent protein) to detect cancer cells among exfoliated cells in urine. This study demonstrates feasibility of analyzing short-term explant cultures isolated from voided urine by means of adenovirus transduction.

## Methods

### Patients and samples

The study protocol was approved by the Institutional Review Board at the Medical University of South Carolina and all patients signed a written consent form before initiation of study participation. Urine samples were obtained by spontaneous micturition from patients with a history of bladder cancer, suspected bladder cancer (hematuria), and volunteers without suspected or confirmed bladder cancer. Eligible patients were >40 years old and had to be free of urinary tract infections at the time of study participation.

### Short-term explant cell cultures

Briefly, urine was collected in a sterile container and centrifuged at 200 x g for 10 min at 22°C. The cell pellet was washed with warm RPMI1640 medium containing 10% fetal bovine serum and centrifuged again at 200 x g. Pellets were resuspended in 4 ml RPMI1640 containing 1% antibiotic-antimycotic solution (Gibco), 1:1000 Primocin (InvivoGen, San Diego, CA) and 20% FBS and were equally divided into 4 wells of a 24-well tissue culture plate (Corning). Plates were incubated at 37°C, 5% CO_2_. Photographs were taken on day 0, on day 3 after media was refreshed and on day 10, when each well was examined for growth. If no growth was observed in any of the 4 wells on day 10, the sample was recorded as "no growth". Outgrowth into a few patches of cells was categorized as "minimal growth" whereas growth throughout the well (sub-confluent or confluent) was categorized as "sufficient outgrowth".

### Cell lines and Cell culture

UM-UC-3 cells were purchased from the American Type Culture Collection (Rockville, MD) and maintained in RPMI1640 medium (Gibco/Invitrogen, Carlsbad, CA) supplemented with heat-inactivated 10% fetal bovine serum (Hyclone, Logan, UT). The UROtsa cell line, kindly provided by Dr. Donald Sens (University of North Dakota, Grand Forks, ND) was derived from normal human urothelial cells and immortalized with the SV40 T-antigen [10]. UROtsa cells were maintained in DMEM medium containing 2 mg/ml glucose and 5% FBS. Cell cultures were maintained at 37°C in a 5% CO_2 _atmosphere (Falcon, Bedford, MA).

### Construction of Ad.Surv.GFP

Dr. Semyon Rubinchik, at the Medical University of South Carolina, generously provided plasmid pShuttle-C.mcs.B. The Sp1 plasmid containing the full-length survivin gene promoter was generously provided by Dr. Maureen Murphy (Fox Chase Cancer Center, Philadelphia, PA) [11]. The pEGFP-C1 plasmid was obtained from Clontech (Mountain View, CA) and was the source of the EGFP gene. Ad.Surv.GFP virus was constructed by ligating the SpeI-Xba1 fragment from Sp1 and the Nhe1-Mlu1 fragment of pEGFP-C1 to form a product with Spe1 and Mlu1 ends. This product was ligated into pShuttle-C.mcs.B digested with MluI and Spe1 to create a shuttle plasmid compatible with the AdEASY adenovirus production system. The completed shuttle plasmid was linearized with PmeI and transformed into competent BJ5183 *E. coli*. Recombinant AdEASY plasmids were purified and transformed into 293 cells to produce virus as previously described [12].

### Analysis of GFP expression

For short-term explant cultures, wells with sufficient growth from a single donor were photographed, trypsinized, combined and redistributed evenly into 3 wells of a 24-well plate. 24 hours later, wells either remained uninfected or were infected with 2 × 10^6 ^IU of virus Ad.CMV-GFP (control) or Ad.Surv.GFP in 1 ml medium. UM-UC-3 and UROtsa cells were plated and infected in parallel at 2 × 10^4 ^cells per well for an approximate multiplicity of infection of 100. Photographs were taken at 24 hours and 72 hours post infection with 10× objective and at 1.5 sec. exposure for fluorescence. UROtsa and UM-UC-3 cells, infected with Ad.CMV.GFP or Ad.Surv.GFP, were also analyzed for GFP. For quantification of GFP-positive cells, wells were trypsinized, cells pelleted and fixed in 3.7% formalin. GFP expression was quantified by the MUSC flow cytometry core facility using a FACSCalibur (BD) and Cell Quest software.

## Results

### Short-term explant culture of exfoliated cells from voided urine

**Figure 1:**
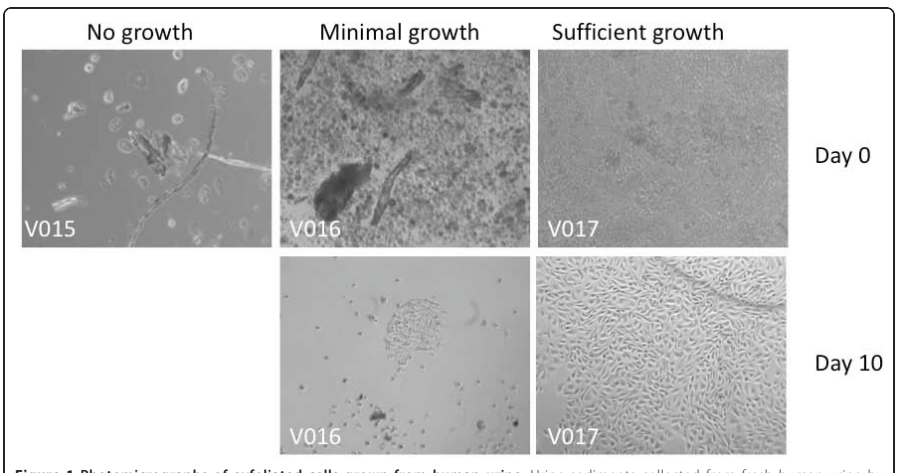
**Photomicrographs of exfoliated cells grown from human urine**. Urine sediments collected from fresh human urine by centrifugation were plated in complete tissue culture medium. Photomicrographs of samples from three donors are shown, taken immediately after plating (Day 0) and 10 days later (Day 10) after a medium change on day 3. (Original magnification, 100X)

Initially 22 voided urine samples were used to optimize parameters for outgrowth and to determine viral infectivity. Samples were from healthy volunteers (no history of bladder cancer or suspected bladder cancer), suspected bladder cancer (presence of hematuria) and history of bladder cancer. Two samples had less than 15 ml volume and three were accidentally stored at 4°C, leading to exclusion from further analysis. Among the remaining samples about half showed evidence of growth (9 of 17, 53%). Microscopic appearance of three representative samples having no growth, minimal growth and sufficient growth is shown in Figure 1.

### Exfoliated cells from urine can be transduced with adenovirus

**Figure 2:**
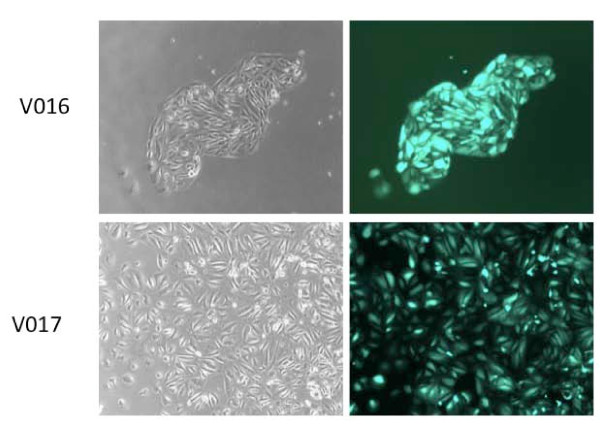
**Adenovirus infection of primary cells grown from exfoliated cells in urine**. Day 16 after plating of urine sediments from two donors, dividing cells were infected with an adenoviral vector expressing green fluorescent protein under control of a cytomegalovirus promoter. Each well of cells was exposed to 2 × 10^6^IU of virus at a concentration of 2 × 10^6 ^IU/ml (Original magnification, 200X)

Transfection of primary cells is generally inefficient and therefore the feasibility of adenoviral-driven reporter gene expression was tested. Two urine short-term explant cultures were exposed to an adenovirus in which a GFP reporter gene is expressed under the control of the CMV promoter. Images captured at 24 hours post-infection demonstrated bright green fluorescence in virtually all cells, indicating that viable, exfoliated cells from urine can be transduced with adenovirus (Figure 2).

### The survivin promoter preferentially drives GFP expression in UM-UC-3 bladder cancer cells when compared to UROtsa cells

**Figure 3:**
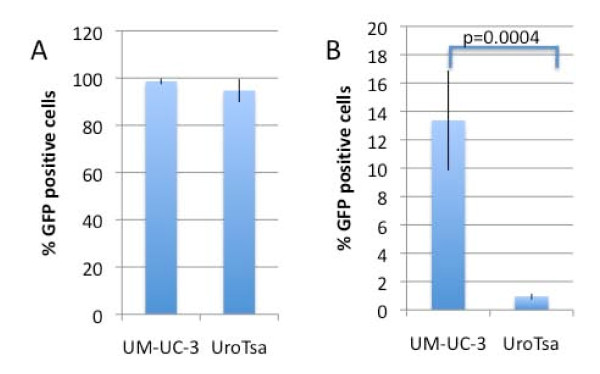
**Transgene expression under survivin and cytomegalovirus promoters in UM-UC-3 bladder carcinoma and UROtsa cell lines**. 50% confluent UM-UC-3 and UROtsa cells (2 × 10^4 ^cells/well) were infected in a 1 ml volume with 6 × 10^5 ^IU of **(A) **Ad.CMV.GFP or **(B) **Ad.Surv.GFP, and analyzed for GFP expression 72 h after infection by flow cytometry. Ad.Surv.GFP expression was corrected for infectivity relative to UM-UC-3 cells, determined for each experiment by Ad.CMV.GFP results. Data shown are the mean ± SD from three independent experiments. P-value was calculated by the student t test.

Having shown that exfoliated cells from voided urine can be used to establish short-term cultures and that these cultures can be efficiently transduced with adenovirus, we generated an adenovirus in which GFP expression was under control of the survivin promoter. GFP expression following Ad.Surv.GFP transduction was quantified in non-malignant UROtsa bladder epithelial cells and the UM-UC-3 bladder cancer cell line. Ad.CMV.GFP served as a control for infectivity. Cells were transduced with Ad.CMV-GFP or Ad.Surv.GFP and GFP expression quantified by flow cytometry at 72 hours post-infection. As shown in Figure 3A, the cell lines transduced with adenovirus equally well. Only a fraction of UM-UC-3 cells were GFP positive when infected with Ad.Surv.GFP indicating that the survivin promoter is substantially weaker than the CMV promoter. Nevertheless, based on GFP expression, the survivin promoter was more active in the UM-UC-3 cancer cell line than in the non-malignant UROtsa cell line (Figure 3B, p = 0.0004).

### Urine exfoliated short-term explant cultures exhibit higher baseline levels of GFP expression than UROtsa cells when infected with Ad.Surv.GFP

**Figure 4:**
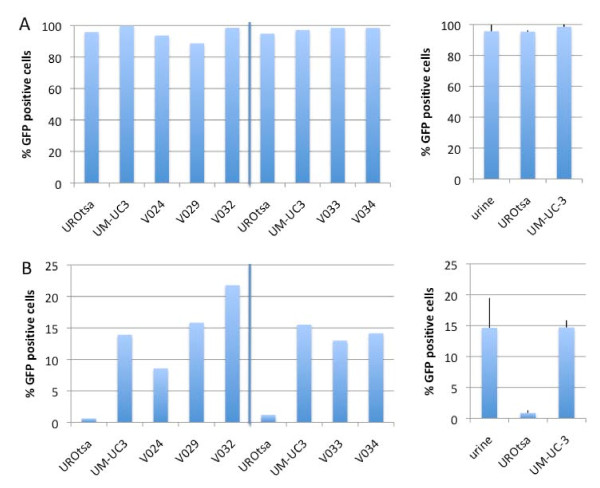
**Transgene expression under survivin and cytomegalovirus promoters in exfoliated cells from urine**. Cell outgrowth from 5 urine samples from healthy controls were infected in two separate experiments with 2 × 10^6 ^IU of virus **(A) **Ad.CMV.GFP or **(B) **Ad.Surv.GFP in 1 ml medium, and analyzed for GFP expression 72 h later by flow cytometry. UM-UC-3 and UROtsa cells were included in parallel in each experiment as controls. Left panels show the raw data from the two experiments, while right panels show the mean ± SD of the three groups.

Next, 14 additional samples collected from healthy volunteers were analyzed for outgrowth and the ability to express GFP from the survivin promoter. Seven samples (50%) displayed growth and of these five had sufficient growth to evaluate viral reporter gene expression following infection with Ad.CMV.GFP or Ad.Surv.GFP. Samples were evaluated in two separate experiments, each including UROtsa and UM-UC-3 as controls. All of the samples were efficiently transduced with Ad.CMV.GFP (Figure 4A). When transduced with Ad.Surv.GFP, UROtsa cells used as a negative control displayed less than 1% GFP positive cells, whereas approximately 15% of UM-UC-3 cells were positive (Figure 4B). Interestingly, the urine exfoliated short-term explant cultures expressed survivin-driven GFP with an efficiency that was similar or higher than UM-UC-3 cells (Figure 4B).

## Discussion

Bladder cancer has a recurrence rate of up to 70% necessitating patients with a history of bladder cancer to undergo years if not lifetime surveillance via periodic cystoscopy, which significantly burdens the healthcare system [13]. There is also no reliable screening test for early detection of bladder cancer. Urinary biomarkers are an active area of investigation and current molecular cytologic tests include ImmunoCyt, which detects the glycosylated form of CEA and mucin glycoproteins (median sensitivity of 81%; median specificity of 75%), DD23 (Median sensitivity of 81%; median specificity of 60%), Lewis X antigen (median sensitivity of 84%; median specificity of 80%), and UroVysion test used to detect aneuploidy for chromosomes 3, 7, 17 and loss of 9p21 locus (median sensitivity of 73%; median specificity of 90%) [14]. Recently, fibroblast growth factor receptor 3 (FGFR3) mutations have been used to detect concomitant recurrences of low-grade non-muscle-invasive bladder cancer with a sensitivity of 58% [15]. While it is apparent that different molecular bladder tumor markers and clinical tests exist to detect bladder cancer [16], these tests do not preclude the recommended invasive and frequent surveillance urethrocystoscopies. Here, we have examined the feasibility of screening short-term explant cultures from voided urine with an adenoviral reporter construct.

A total of 31 samples were evaluated for the ability to grow short-term explant cultures from voided urine. In agreement with a recent study we found that cell attachment and propagation occurred within 10 days after culture initiation [17]. About half of all samples (16/31) showed evidence of *in vitro *growth. This included 4/6 samples from patients with a previous history of bladder cancer (all white males with a median age of 69.5, 72.7 +/- 10.4), 4/5 samples from patients presenting with hematuria (1 WF, 2BF, 1WM, 1BM, median age 64, 60.8 +/- 14.7) and 8/20 healthy volunteers (8WM, 2BM, 10WF, 1AF, median age 52.5, 52.9+/- 9). In a previous study, primary culture outgrowth from urine, defined as the presence of islet-like cells, was observed in 54.7% of healthy volunteers and in 86.6% of bladder cancer patients [18]. Since none of our samples were from patients currently having bladder cancer, our rate of 52% outgrowth is similar to the previously observed 54.7%. Upon closer examination however, the outgrowth rate from healthy volunteers approached 90% (7/8) in samples that were processed within 30 minutes of urine collection. These results suggest that a short time to processing is a critical factor to successfully establish short-term cultures from voided urine.

Our results also demonstrate that exfoliated cells obtained from spontaneous micturition can be transduced with adenoviral reporter vectors, which provides a novel opportunity to detect malignant cells via a non-invasive screening modality. Survivin, a member of the inhibitor of apoptosis family of proteins involved in regulating cell division and apoptosis, is overexpressed in tumor cells relative to normal cells. Therefore, the survivin-promoter has been used to transcriptionally target tumors for gene therapy [9], including delivery of adenovirus-based vectors and conditionally replicative adenoviruses in treating malignant gliomas [19,20]. Survivin mRNA was detected in urine with 94% sensitivity and 95% specificity [21], suggesting the survivin promoter is preferentially activated in bladder cancer cells. In this study we used the full-length survivin promoter to drive GFP reporter gene expression in normal and malignant bladder cancer cells. Non-malignant UROtsa and malignant UM-UC-3 bladder cells were used to test the survivin-driven reporter gene expression. As expected, CMV-driven GFP expression was similar between the cell lines whereas survivin-driven GFP expression was preferentially observed in the malignant UM-UC-3 cells (Figure 3).

However, when short-term explant cultures obtained from urine of healthy volunteers were infected with Ad.Surv.GFP, the percentage of GFP positive cells was comparable to the UM-UC-3 positive control rather than the non-malignant UROtsa cells, which served as a negative control (Figure 4). There are several possible explanations for this observation. Survivin has dual roles in regulation of G1/S transition and apoptosis protection [22]. Thus one possibility is that the level of survivin promoter activity in the explant cultures is dictated by proliferation and progression through the cell cycle. However, we did not observe the growth of explant cultures to be more rapid than UROtsa cells, which would argue against proliferation as a cause for higher GFP expression following Ad.Surv.GFP expression. While it has been widely accepted that survivin is expressed at low levels, if at all, in normal differentiated tissues, there are also reports of survivin expression in normal breast tissue and fibroadenomas [23]. Weikert et al. have shown that survivin is expressed in human testicular germ cell tumors as well as in human normal cells of the testes [24]. These studies suggest that baselines of survivin expression and/or promoter activity have to be established for each cell line or primary culture. Thus our data may indicate that primary cultures established from voided urine simply exhibit a higher basal level of survivin promoter activity than established bladder cell lines.

## Conclusions

In conclusion, our study demonstrates that short-term explant cultures can be established from at least half of spontaneously voided urine samples and at higher rates if processing time does not exceed 30 minutes. Sufficient cells can be obtained for adenoviral transduction and quantification of GFP reporter activity by flow cytometry. Our feasibility study is limited by the subject population, necessitating a future larger study to determine baseline level of Ad.Surv.GFP expression in explant cultures from healthy volunteers. In addition, spontaneously voided urine from patients with bladder cancer will need to be included to determine specificity and sensitivity of the non-invasive Ad.Surv.GFP test.

## Competing interests

The authors declare that they have no competing interests.

## Authors' contributions

AM and LK prepared cultures and conducted the experiments. In addition LK generated the Ad.Surv.GFP and AM drafted the manuscript. CVJ conceived of the study and participated in its design and coordination and edited the manuscript draft. All authors read and approved the final manuscript.

## Pre-publication history

The pre-publication history for this paper can be accessed here:

http://www.biomedcentral.com/1471-2407/11/168/prepub
